# Modeling for a Smallpox-vaccination Policy against Possible Bioterrorism in Japan: The Impact of Long-lasting Vaccinal Immunity

**DOI:** 10.2188/jea.14.41

**Published:** 2005-03-18

**Authors:** Hiroshi Nishiura, I. Ming Tang

**Affiliations:** 1 Bangkok School of Tropical Medicine, Mahidol University, Thailand.; 2 Department of Mathematics, Faculty of Science, Mahidol University, Thailand.; 3 Institute of Science & Technology for Research and Development, Mahidol University, Thailand.

**Keywords:** smallpox, bioterrorism, models, mathematical, vaccination, immunity

## Abstract

BACKGROUND: There has been concern that variola virus might be held clandestinely elsewhere. Through constructing mathematical model based on the detailed epidemiologic data, we focused on simulating the various possible scenarios arising from a bioterrorist attack whereby smallpox virus was introduced into Japan, and sought to develop the most effective way of nationwide vaccination policy based on the theory of residual immunity.

METHOD: The analysis is based on a deterministic mathematical model which predicted the epidemiologic outcome while simultaneously evaluating the effect of any specified control strategy of the smallpox epidemic. To clarify the required amount of vaccines, we performed mathematical analysis for hypothetical population to acquire herd immunity based on long-lasting vaccinal immunity.

RESULTS: It is demonstrated that the crude size of the potential epidemic could be greatly affected by possible level of residual immunity. The results also suggest the possibility to develop optimal distribution of nationwide vaccination according to the immune status. The prevalence at 50th day among population without immunity in our simulation would be approximately 405 times greater than expected population with residual immunity, and required amount of vaccines for equal distribution would be 3.13 times more than optimal distribution.

CONCLUSION: The mathematical model formulated could determine the vaccination priority based on the real status of immunity which required much less amount of vaccinations than would be calculated using an equal distribution program. It is therefore crucial to determine the real immunity status of the population via epidemiologic studies.

Bioterrorism is the intentional use of micro-organisms, or their products, to cause harm, and may be used to target humans, animals or crops.^1^ Variola virus, which causes smallpox, is one of the most dangerous bioterrorism agents to be worried about. If used as a biological weapon, it poses a serious threat to the civilian populations because of its case fatality proportion of 30% or more among the unvaccinated persons and the absence of specific therapy.^2^ Furthermore, because the World Health Organization (WHO) announced the total eradication of the smallpox in 1979, routine vaccination gradually ceased worldwide,^3^ leaving the younger age individuals today who have never been vaccinated, and are thus extremely susceptible to smallpox infection. There is concern that the virus might be held clandestinely and less securely elsewhere.^4^ In the aftermath of the September 11 terrorist attacks in 2001, the United States, after receiving direct threats, began stockpiling 286 million doses of smallpox vaccine, and the Centers for Disease Control and Prevention (CDC) interim response plan calling for targeted vaccination and quarantine.^5^ Disaster plans for managing a biological attack must be developed in detail and realistic training provided to ensure effective response to an actual terrorist event.^6^

Japan should not feel that it is exempt from the various terrorist threats. It has been said that if Japan were to become a key ally in a United States-led military campaign against terrorism in Asia, both Japan and Japanese living abroad would become terrorist targets.^7^ In response to the mailborne anthrax terrorist attacks in the United States,^8^ the Ministry of Health, Labor and Welfare of the Japanese government has formed a working group on protection against bioterrorism in December 2001, and has started preparing mass production of vaccine against smallpox for 10 million civilian persons using less neuropathogenic tissue culture freeze-dried vaccine with LC16m8 strain.^9^ The Ministry has also begun to prepare vaccines for first-line health care workers in case of smallpox bioterrorism. The Self Defense Force personnel who are serving in peace-keeping operations in the Middle Eastern countries have already been vaccinated.^9^ Although the Ministry has announced a contingency plan for a possible outbreak of smallpox in Japan,^10^ detailed information and guidelines are still lacking when compared to the ones produced by the CDC. Although the Japanese government prepared its plan using much from the CDC, unlike CDC, it has neither given the scientific justification in their policy for amount of the vaccines necessary, nor provided the reason why post-exposure vaccination should be carried out within the four days after exposure. Because the Japanese government has not made it clear to the public its policy and intention regarding the smallpox vaccination, the public until date remains ignorant and thus unprepared.

In the face of many unknowns, several mathematical epidemiologists have challenged the presently used models for assessing public health interventions including the vaccination policy regarding the survival and spread of smallpox,^11^^,^^12^^,^^13^^,^^14^ or for estimating its transmissibility using past epidemiologic records.^15^^,^^16^ Models may be conceptualized as thought experiments, and are extremely useful tools when physical experiments are impossible to perform due to time, monetary, practical, or ethical constraints.^17^. The purposes of this study are to simulate the possible scenarios which could arise from a bioterrorist attack of introducing smallpox into Japan, and to describe the possible outcome of different nationwide vaccination policies based on the hypothesis on residual immunity in the population. This would allow the Japanese government to impose its original vaccination policy, and determine what new epidemiologic study is needed.

## METHODS

### Mathematical Model

**Figure 1.**
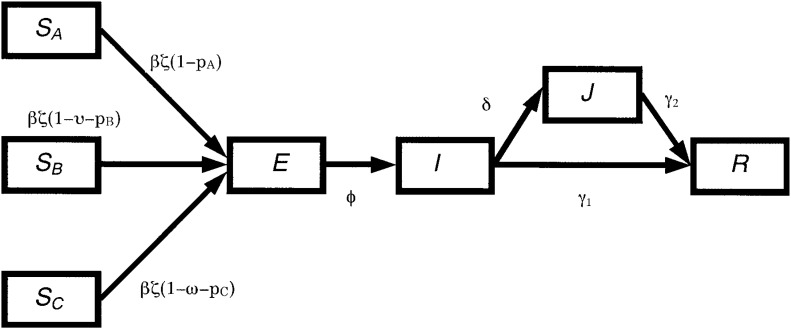
The transmission dynamics of the smallpox taking into account the impact of different residual immunity and interventions. Here: *S_A_*, *S_B_* and *S_C_* represents the proportion of population susceptible among Groups A (born after 1977), B (born in 1969-1977), and C (born before 1969), respectively; *E* represents the proportion of untraced latent individuals; *I* the proportion of the population infectious; *J* the proportion of infectious isolated; *R* the proportion of recovered and death.

The analysis presented in this paper is based on a deterministic mathematical model for epidemic which could predict the epidemiologic outcome while simultaneously evaluating the effect of any specified control strategy on smallpox. The model is a modification of the *SEIJR* model,^18^ which separates the population into the classes of people who are susceptible (*S*), exposed (*E*), infectious (*I*), diagnosed (*J*), and recovered (*R*). The model is described by a set of ordinal differential equations which are based upon specific biological and intervention assumptions about the transmission dynamics of smallpox (Figure 1). We first separate the susceptible population (*S*) into three age groups according to the expected immunity:^19^ (Group A) represents those who have never been vaccinated (*S_A_*) i.e., born after 1977, and who constitute the proportion (*1-x-y*) of the total population, (Group B) represents those who received only primary vaccination (*S_B_*) i.e., born between 1969-1977, and is denoted as *xN*, and (Group C) represents those who have received both primary and revaccination (*S_C_*) i.e., born before 1969, and is denoted as *yN*. A certain proportion from each population groups (A, B, and C) are assumed to be effectively protected by present vaccination strategy, and denoted as *p_A_*, *p_B_* , and *p_C_*. A smallpox infection among the susceptible population (*S_A_*, *S_B_*, and *S_C_*) firstly begins with a non-infectious incubation period (*E*), which constitute the latency period. It would be followed by prodrome with non-specific symptoms, and by an overtly infectious (*I*) and symptomatic stage, characterized by a pustular rash. By this time, most of infections would be apparent and be diagnosed (*J*). The patients would then either slowly recover or die (*R*).^20^ While infectious, the infected patients can transmit the disease to other susceptible individuals at a rate dependent on the basic reproduction number, *R_0_*.^21^ There are currently three possible public health interventions for interrupting the transmission of the virus. These are (1) vaccinating those who are at risk or may have already been exposed, (2) quarantining certain proportion of those who are known to have been exposed and therefore may be infected but are not yet ill (see Appendix), and (3) moving infectious individuals (*I*) into isolation after being diagnosed (*J*). We assume that each susceptible makes *ζ* contacts per day with an infectious person. Among the known contacts (in *S_A_*, *S_B_*, and *S_C_*), some would be infected with the probability of *β* per contacts (and enter into *E*) and (*1-β*) remains uninfected and susceptible. Untraced infectious persons would recover or die after (*γ_1_*)^−1^ days. Apparent infectious person would be diagnosed and isolated with the mean daily rate *δ* (and enter into *J*), and recover or die (*γ_2_*)^−1^ days after isolation. Because isolation can never be perfect, we estimate that those who are isolated also contribute to the generation of newly infected cases. Therefore, relative measure of reduced risk among those isolated (*π*) is multiplied to *J*. These processes can be modeled using an approximately parameterized set of differential equations [1] as given by:

$$\begin{array}{rl} \frac{dS_{A}}{dt} & =-\zeta \beta (1-p_{A})S_{A}(I+\pi J)\\ \frac{dS_{B}}{dt} & =-\zeta \beta (1-\upsilon -p_{B})S_{B}(I+\pi J)\\ \frac{dS_{C}}{dt} & =-\zeta \beta (1-\omega -p_{C})S_{C}(I+\pi J)\\ \frac{dE}{dt} & =-\beta \zeta \{(1-p_{A})S_{A}+(1-\upsilon -p_{B})S_{B}+(1-\omega -p_{C})S_{C}\}(I+\pi J)-\phi E\\ \frac{dI}{dt} & =\phi E-(\gamma_{1}+\delta )I\\ \frac{dJ}{dt} & =\delta I-\gamma_{2}J\\ \frac{dR}{dt} & =\gamma_{1}I+\gamma_{2}J \end{array}$$ [1]

Because isolation measure is not usually undertaken in the early stage of the epidemic (the stage of which is given by *S_A_+S_B_+S_C_ = N* and *(E(t), I(t), J(t)) = (0, 0, 0)*, where *N* is the size of the population in which the epidemic occurs), *I(t)* at the initial attack without the effect of quarantine would be given by:

$$\frac{dE(t)}{dt}=[\beta \zeta \{(1-p_{A})S_{A}(0)+(1-\upsilon -p_{B})S_{B}(0)+(1-\omega -p_{C})S_{C}(0)\}-\gamma_{1}]I(t)$$ [2]

Therefore, the growth of infectious person at initial stage will follow Malthusian model as follows:

$$I(t)\approx I(0)e^{[\beta \zeta \{(1-p_{A})S_{A}(0)+(1-\upsilon -p_{B})S_{B}(0)+(1-\omega -p_{C})S_{C}(0)\}-\gamma_{1}]t}$$ [3]

From the second generator approach,^22^ we obtain the following expression for the basic reproduction number, *R_o_*:

$$\begin{array}{rl} R_{0} & =\zeta \beta N\{(1-p_{A})(1-x-y)+(1-\upsilon -p_{B})x+(I-\omega -p_{C})y\}\\ & \;\times \left\{\frac{1}{\delta +\gamma_{1}}+\frac{\delta \pi }{\gamma_{2}(\delta +\gamma_{1})}\right\} \end{array}$$ [4]

A description of the other principal parameters in the model and of their assigned value is presented below.

### Parameter Values

Table 1 contains the parameter values for our baseline case. Assuming that the biological variables do not differ much from those of past epidemics, we use the values given in previous epidemic modeling studies^12^^,^^13^^,^^15^^,^^16^ for possible scenario analyses. The infection rate *βζ* is chosen and fixed so that *R_0_* = 6.87, which is derived from an estimate on the order of 4.52 to 10.1 estimated in the previous study that have involved calculation of *R_0_*.^16^ Because our purposes here are to draw crude pictures of the possible smallpox bioterrorist attack and describe the impact of residual immunity, we performed an analysis based on a single value of *R_0_* (although we have varied parameter assumption in sensitivity analysis for assumed residual immunity and initial attack size discussed below). These types of analyses on the impact of public health interventions are beyond the scope of this paper. Such studies have already been undertaken elsewhere.^11^^,^^12^^,^^13^^,^^14^ Therefore, our simulation itself in this paper excludes the effect of quarantine (see Appendix, where we formulated the model incorporating the effect of quarantine). We assume that the pattern of contact is linearly related to the population size so that every infectious person will pass the disease to exactly *R_0_* susceptible individuals simultaneously within an incubation period of (*ϕ*)^−^*^1^* days. From this assumption, *ζN* denotes the daily number of contacts in the population. In addition, we use a single value of *R_0_* throughout the epidemic that represents the post-detection scenario so as to estimate the natural course (without interventions) of the epidemic although the transmission rate is likely to decrease after the epidemic is detected and announced. Although homogenous (or free) mixing is not an accurate description of the actual population interactions, free mixing usually leads to larger epidemics than nonrandom mixing.^23^ In addition, we assumed homogenous mixing because smallpox infections as caused by a bioterrorist attack would not necessarily accumulate in a small number of limited locations. We start the bioterrorism scenario with an entry of 10 initial cases into a population of 1,000,000 people, with enough population density to give the more than necessary critical proportion of the population, as our baseline case. It is assumed that one million people with a certain population density is a typical representation of a population of one ward in an urban area in Japan (i.e., Setagaya ward of Tokyo has a population of 815,000). It is somewhat unrealistic to expect the population at the prefectural or national level to be at risk because it would not be possible to have 100% of this population to come into possible direct or indirect contact with the disease within the short time period of concern. We have therefore considered a scenario of an epidemic in a city or ward sized population, such as the one of Setagaya.

**Table 1.** Parameter values for transmission dynamics of smallpox.

Parameters	Description	Baseline Values	Referrence
*β*	The probability of transmission per contacts	*βζ* = 4.26	*a

*ζ*	The daily number of contacts per capita		*a

*p_A_*	The proportion of exposed person among Group A who was effectively protected by vaccination	0.00	*b

*p_B_*	The proportion of exposed person among Group B who was effectively protected by vaccination	0.00	*b

*p_C_*	The proportion of exposed person among Group C who was effectively protected by vaccination	0.00	*b

*ϕ*	The average rate at which latent individuals become infectious	0.0685 day^-1^	3

*δ*	The mean daily rate at which infectious cases are diagnosed and isolated	0.95 day^-1^	3

*γ_1_*	The percapita rate for recovery and deth	0.116 day^-1^	27

*γ_2_*	The percapita rate for recovery and deth after isolated	0.132 day^-1^	3,28

*π*	Relative measure of reduced risk among isolated cases	0.10	16,29

*x*	The proportion of Group B population	0.105	19

*y*	The proportion of Group C population	0.601	19

*υ*	The proportion of population with residual immunity among Group B estimated	0.30	19,24

*ω*	The proportion of population with residual immunity among Group C estimated	0.90	19

*a: The infection rate *βζ* is chosen and fixed so that the basic reproduction number becomes 6.87.

*b: Projected epidemic curves (baseline case) given by simulation ignored vaccination.

Discussion for these parameters are given in text.

We first simulate three possible scenarios for different proportion of people whose residual immunity still exist. In the first scenario, based on the hypothetical long-lasting immunity in Japan^19^ estimated using latest study in India,^24^ we assume that approximately 30% of Group B and 90% of Group C (with population size of 1.05 × 10^6^ and 6.01 × 10^6^, respectively) will still have protective immunity against smallpox. The proportion of people with immunity in Group B (*υ*) and C (*ω*) would thus be set as 0.30 and 0.90, respectively. Because Group A consists of only those who were born in 1977 and thereafter have never been vaccinated, no one in this group will have the protective immunity. For the second scenario, we assume that half of estimated population still possesses immunity (*υ=0.15, ω=0.45*). It is believed that loss of immune protection might contribute to the epidemic.^16^ For the third scenario, we assume that no person possess protective immunity (*υ=0, ω=0*). To make the differences between those three scenarios clearly visible, we assume that there is no public health intervention except isolation in all three scenarios.

We then consider the impact of different levels of vaccine distributions for the three age groups (A, B, and C), which would become crucial if nationwide mass-vaccination is required (level III). By estimating the optimal condition in order to prioritize, we generalize the condition with simple mathematical formula so that it can be applied to other communities having different age distribution. Finally, we estimate the total amount of smallpox vaccines needed in Japan using a generalized formula. In this study, the total number of people in the population is assumed to be constant during the epidemic. The background mortality rate is assumed to be negligible over the time periods examined.

### Sensitivity analysis

Because model parameters regarding the proportion of the population in Groups B and C with residual immunity (*υ*, *ω*) and initial attack size (*I(0)N*) possess the most uncertainty, a sensitivity analysis comparing the reproduction number is performed for different settings of them. Firstly, we compare the sensitivity of the reproduction number for either *υ* or *ω*, and then varied both. In three of the comparisons, both *υ* and *ω* are varied from 0 to 1.0 separately. When we vary both of them, we multiplied the relative reliability, which we define as a variable from 0 to 1.0, to our assumed immune proportion (*υ* = 0.30 or *ω* = 0.90). As for the initial attack size, we analyzed the reproduction number by varying *I(0)N* from 10 to 100,000 cases. A hundred thousand is selected as the maximum number of initial cases because it would be 10% of the total population. Whatever the way of introduction would be, we consider it is unrealistic to assume much more number of initial cases in our assumed ward-sized community.

## RESULTS

**Figure 2.**
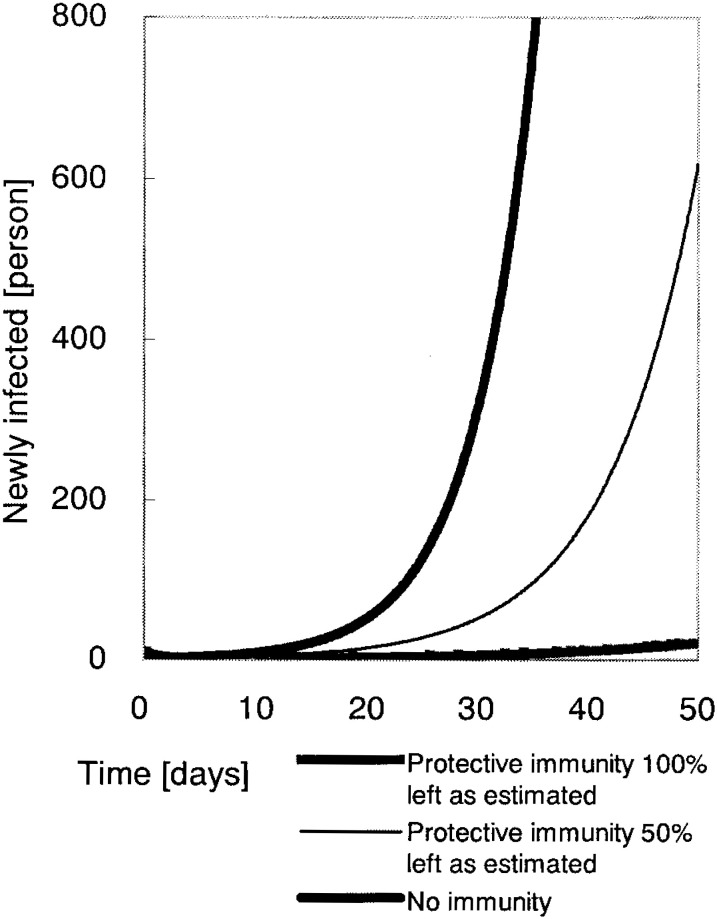
Dynamics of a smallpox attack with the basic reproduction number *R_0_* = 6.87. The number of infectious smallpox cases according to the protective (residual) immunity in Groups B (born in 1969-1977) and C (born before 1969), *υ* and *ω*. Simulations were performed with a time-step of 0.1 days.

The result of a simple scenario analysis is seen in Figure 2. It shows the probable dynamics of the smallpox epidemics under different conditions of residual immunity. The results are given for up to 50 days after the onset of epidemic. It is unrealistic to estimate for longer period of time because one would not expect the health policy and control strategies as well as social reactions to remain static over longer periods. Without any public health interventions and protective immunity, exponential growth of daily number of new cases would occur. The point prevalence (here denoted as the number of infectious individuals) would exceed 500 persons by the 33rd day after onset of epidemic. If the half of estimated immune population in Groups B and C still possesses immunity, the incidence rate (=rapidity) of smallpox will be lessened, but the trend of exponential growth would not cease without any interventions. On the other hand, the daily number of new cases would be in relatively controllable number if parts of the Groups B and C were perfectly immune as hypothesized. It is notable that trend of increase would still be observed without interventions. The difference in the prevalence between a population which had no immunity and the one which had the expected immunity at 50th day would be approximately 405 folds.

If a proportion p of the population is successfully immunized, the critical proportion of the population to be immunized (*p_cri_*), which is needed to attain the eradication,^21^ is given simply by:

$$p_{\text{cri}}=1-\frac{1}{R_{0}}$$ [5]

Approximate estimate of the vaccination coverage (the degree of herd immunity) needed to eradicate smallpox is known to be in the order of 70 to 80%.^21^ Here, we separate the susceptible into three age groups based on their possible residual immunity. Based on this assumption, the condition to break the chain in the person-to-person transmission of smallpox is given by the equation [4]:

$$(1-p_{A})(1-x-y)+(1-\upsilon -p_{B})x+(1-\omega -p_{C})y<\frac{1}{R_{0}}$$ [6]

where *x* and *y* are the proportion of Groups B and C in susceptible population, respectively. Population of the Group A can be represented as (*1-x-y*)N. If the hypothesized level of immunity were perfectly realistic (such as our baseline case, *υ* = 0.30 and *ω* = 0.90 described in Table 1), the prioritization in order to achieve the most effective vaccine intervention can be calculated by:

$$\begin{array}{rl} f(p) & =(1-x-y)p_{A}+xp_{B}+yp_{C}\\ & =0.294p_{A}+0.105p_{B}+0.601p_{C} \end{array}$$ [7]

According to Arita’s assumptions,^19^ which he calculated from another study carried in India,^24^ the optimal distribution of vaccine priority should be based on the population without immunity:

$$\begin{array}{rl} m_{A}:m_{B}:m_{C} & =(1-x-y):(1-\upsilon )x:(1-\omega )y\\ & \;;69:17:14 \end{array}$$ [8]

where *m_A_*, *m_B_*, and *m_C_* are the ratio of the population who have not been immune based on residual immunity by Group A, B, and C. When we assume that the total amount of vaccines would be constant (for the purpose of comparison of immune population to be covered), we can transform these conditions into the ratio of proportion in need of vaccination by adjusting for the number in each population as,

$$p_{A}:p_{B}:p_{C};86:9:5$$ [9]

The conditions here are expressed as the proportion of people who needs to be vaccinated in each age group. Based on the equation [6], the optimal distribution of vaccine priority (the amount of vaccines) should be:

$$v_{A}:v_{B}:v_{C};84:4:12$$ [10]

where *v**_A_*, *v**_B_*, and *v**_C_* are the amount of vaccines needed by Group A, B, and C. Because we have set *R_0_* = 6.87, into eqn. [6] and [7], the minimum coverage and amount required to cause the smallpox epidemic to settle down in each age group is,

$$\begin{array}{rl} p_{A}\geq 74.21\% & \;v_{A}\geq 218,169\\ p_{B}\geq 2.65\% & \;v_{B}\geq 2,783\\ p_{C}\geq 8.59\% & \;v_{C}\geq 51,605 \end{array}$$ [11]

The total amount of vaccination (*V*) should cover at least *v**_A_*+*v**_B_*+*v**_C_* = 272,557 persons in this scenario analysis. This can be calculated from:

$$\begin{array}{rl} V & \geq v_{A}+v_{B}+v_{C}\\ & =\{p_{A}(1-x-y)+p_{B}x+p_{C}y\}N \end{array}$$ [12]

Approximate coverage should be more than 27.3% of total population in our scenario analysis, while it would be necessary to cover 85.4% if we do not take immunity into account. If it becomes necessary to carry out nationwide mass-vaccination, we would need vaccines for between 27.3 and 85.4 million people depending on the different policies. Because the number does not take efficacy of the vaccine into account, the actual coverage might be greater than is given here.

**Figure 3.**
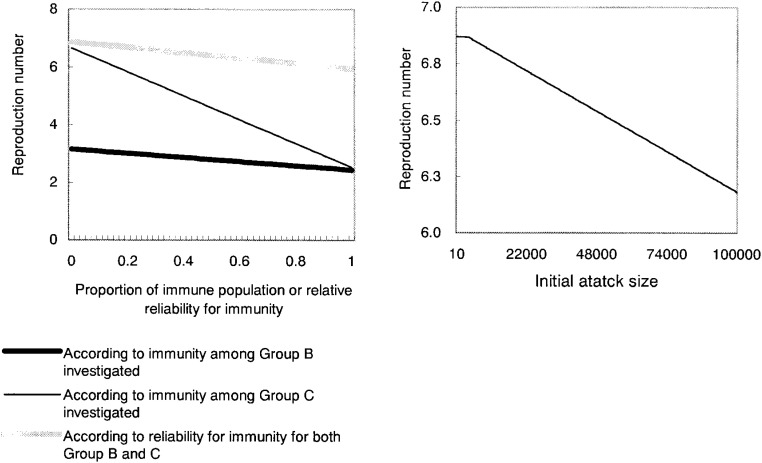
Sensitivity analysis for uncertain parameters. The reproduction number under (a) varied proportion of the population with residual protective immunity as well as relative reliability for immunity among the population in Groups B (born in 1969-1977) and C (born before 1969), (b) different initial attack sizes from 10 to 100,000. The total population size was fixed at 10^6^.

Figure 3 shows the results of sensitivity analysis. The reproduction number changes linearly related to *υ*,*ω* and *I(0)N*. Comparing the proportion of the population possessing residual immunity between Group B and C, the reproduction number by varying Group B is more sensitive than C to the proportion of the immune population. Although the reproduction number increases as the relative reliability declines for both *υ* and *ω*, its increase seems rather small compared to drastic change in *ω*. The reproduction number will also decline when initial attack size increases. However, compared to the change of the reproduction number on the order of 2.4 to 6.9 in Figure 3 (a), the varying interval in Figure 3 (b) is limited such as from 6.1 to 6.9

## DISCUSSION

Two important conclusions can be drawn from our assessments of the impact of immunity on possible smallpox epidemic in Japan. First, it demonstrates that the crude size of the potential epidemic could be greatly affected by the possible residual immunity within the population. Depending on the actual protective immunity, huge differences in smallpox incidence among the various population groups might be observed. Secondly, it is possible to determine how the optimal levels of vaccination should be when a nationwide vaccination becomes necessary, which is based on the immune status of the individuals. Therefore, if we could formulate a prioritization scheme for vaccination, which is based on the immunity of the individual, the total amount of vaccines could turn out to be much lower than the estimate given by the equal distribution policy.

Despite the problems of uncertainty with the real epidemiologic data of bioterrorism, a simple dynamic model still gave reasonable simulations of the smallpox dynamics. Because transmission potential varies from community to community, we performed a sensitivity analysis according to the residual immunity and initial attack size for determined *R_0_*, which was within the range of precise estimate. Because initial attack size itself does not largely affect the transmission potential, the size of epidemic would be linearly increase according to the initial attack size in further simulations based on our assumption (mostly it originates from assuming homogenous mixing). Although the results of a long-lasting protective effects of smallpox vaccination is still based on theoretical analysis^25^ with certain assumptions, the impact of residual immunity on the size of epidemic can clearly be demonstrated when we examine the natural course of epidemic (without any interventions). Because mass-vaccination measures greatly affect the transmissibility, the rapidity with which the smallpox epidemic spread would largely be lessened by the presence of the residual immunity. The national policy to achieve optimal distribution of vaccination should therefore be formulated on the basis of residual immunity among its population. This might affect the longevity of the epidemic as well as how fast it spreads. The contribution of residual immunity to the probability of controlling smallpox outbreak can be described by equation [6]. It might be possible to estimate the total amount of vaccines needed (equation [12]) when smallpox begins to spread into each community. The model has also been generalized so that it can be used to achieve the estimation for other communities. The minimum amount of vaccines that needs to be stocked in order to obtain herd immunity (or eradicate) against smallpox would be 3.13 times lesser than the amount needed when no immunity exists. According to sensitivity analysis, the possible trajectories would be sensitive to the proportion of immune population in Group C. It is considered to be due to the large number of the population in Group C. The overall number of vaccine doses would be an underestimate because efficacy of the vaccine must also be taken into account if a mass-vaccination was to take place.

Although our study demonstrates the large impact of residual immunity on the epidemic, the real percentage and duration of immunity are unknown. Our study is based on certain assumptions. It is therefore critically important to know the status of immunity in the real population from epidemiologic studies. In this study, we considered the impact of varying residual immunity in each age group by looking at the sensitivity of associated parameters (*υ* and *ω* being the most critical). Such sensitivity analyses can help estimating the variability in the size of epidemic and the reproduction number. One should also note that mass vaccination before a bioterrorist attack actually takes place is not practicable in the real settings. Although we focused on the impact of residual immunity and its application for calculating required stock for vaccination as a possible implication, vaccination would start after an identification of the attack. There would be a race of time between implementing of vaccination and the spread of transmission.^12^^,^^26^ For the purpose of practical planning or simulation, it would be necessary to consider these important aspects.

Our study has several limitations, however. Much needs to be overcome in order to increase model realism. First, one of the major problems, which the world must confront, is the uncertainty and lack of knowledge on smallpox bioterrorism. We believe that one approach to overcome the problem of risk management is to model the potential episodes with mathematical modeling. This study was conducted with only a few known parameter values, and our method assumed a closed population with crude results (in addition, simulations without quarantine). Although we assumed the introduction of smallpox into an urban community, epidemic could be different between urban and rural communities because population density as well as many of the socio-demographic and behavioral characteristics vary.^30^ Thus there are many uncertainties. It should be noted that many other variables could affect the course of epidemic in real world bioterrorism such as the pattern of contacts. Although we assumed homogenous mixing within the community, the spread in scale-free networks should further be considered, and intercommunity migration should also be taken into account. Secondly, the estimation of total amount of vaccine needed is based on optimistic assumption. We do not know the actual percentage of residual immunity and the vaccine efficacy. We need further epidemiologic studies on immunity as well as on vaccine trials for smallpox. Finally, although possible outcomes were determined for a certain population size, one should not expect the same outcome for cities of the same size because of regional variances in the age distribution. Since the formula for the total amount of vaccination needed has been generalized, each community should be able to calculate the requirements based on their own epidemiologic records and age distributions. In order to prepare the various communities, including ours, for future possible bioterrorist attacks as well as to facilitate the use of mathematical models in policy formulation, we open ourselves to criticisms, comments and suggestions for collaborations with others academic who share the same concern.

## Figures and Tables

**Figure 4.**
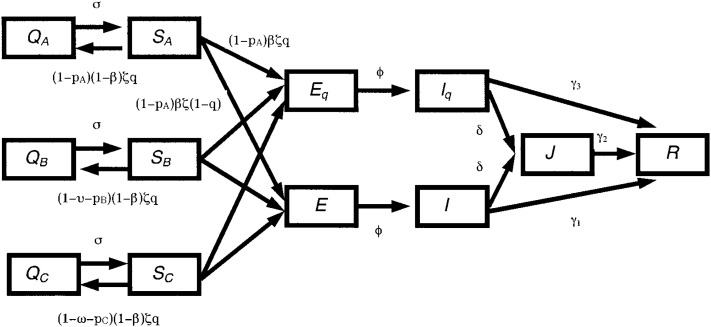
The transmission dynamics of the smallpox incorporating the effect of quarantine. Additional compartments: *E_q_* represents the proportion of traced latent contacts; *Q_A_*, *Q_B_* and *Q_C_*, the proportion of traced uninfected contacts from Groups A (born after 1977), B (born in 1969-1977), and C (born before 1969); *I_q_* the proportion of infectious in quarantine.

## APPENDIX

For the purpose of further realistic simulation, here we consider the effect of quarantine onto our model (using additional five compartments, Figure 4). We suppose the fraction *q*, of those who are known to have been exposed and therefore may be infected but are not yet ill, would be quarantined (denoted by the compartment *E_q_*). Those who are in *E_q_* will become infectious after the latency period (and enter into *I_q_*). Some of them would be diagnosed and moved into isolation with the mean daily rate *δ* (and enter into *J*). The other of infectious and traced individuals are assumed to recover or die after (*γ*_3_)^-1^ days of quarantine. In addition to quarantining the infected individuals, we need to consider uninfected and traced individuals. Among uninfected, *(1-q)(1-β)* remains susceptible and *q(1-β)* would be traced and enters into *Q_A_*, *Q_B_* and *Q_C_*: which represent those who are traced but uninfected for each age group. Those who were traced but uninfected would finish quarantine (released into community again and enter the susceptible population) *σ*^-1^ days after their known contact. Since we should assume the quarantine can never be perfect to protect an additional transmission, relative measure of reduced risk among those quarantined (*θ*) is multiplied to *I_q_*. Incorporating these assumptions onto equations [1], the transmission dynamics with the modification of quarantine system can be described to

$$\begin{array}{rl} \frac{dS_{A}}{dt} & =-\zeta (1-p_{A})\{q+\beta (1-q)\}S_{A}(I+\theta I_{q}+\pi J)+\sigma Q_{A}\\ \frac{dS_{B}}{dt} & =-\zeta (1-\upsilon -p_{B})\{q+\beta (1-q)\}S_{B}(I+\theta I_{q}+\pi J)+\sigma Q_{B}\\ \frac{dS_{C}}{dt} & =-\zeta (1-\upsilon -p_{C})\{q+\beta (1-q)\}S_{C}(I+\theta I_{q}+\pi J)+\sigma Q_{C}\\ \frac{dQ_{A}}{dt} & =(1-p_{A})(1-\beta )\zeta qS_{A}(I+\theta I_{q}+\pi J)-\sigma Q_{A}\\ \frac{dQ_{B}}{dt} & =(1-\upsilon -p_{B})(1-\beta )\zeta qS_{B}(I+\theta I_{q}+\pi J)-\sigma Q_{B}\\ \frac{dQ_{C}}{dt} & =(1-\omega -p_{C})(1-\beta )\zeta qS_{C}(I+\theta I_{q}+\pi J)-\sigma Q_{C}\\ \frac{dE_{q}}{dt} & =\beta \zeta q\{(1-p_{A})S_{A}+(1-\upsilon -p_{B})S_{B}+(1-\omega -p_{C})S_{C}\}(I+\theta I_{q}+\pi J)-\phi E_{q}\\ \frac{dE}{dt} & =\beta \zeta (1-q)\{(1-p_{A})S_{A}+(1-\upsilon -p_{B})S_{B}+(1-\omega -p_{C})S_{C}\}(I+\theta I_{q}+\pi J)-\phi E\\ \frac{dI_{q}}{dt} & =\phi E_{q}-(\gamma_{3}+\delta )I_{q}\\ \frac{dI}{dt} & =\phi E-(\gamma_{1}+\delta )I\\ \frac{dJ}{dt} & =\delta (I+I_{q})-\gamma_{2}J\\ \frac{dR}{dt} & =\gamma_{1}I+\gamma_{2}J-\gamma_{3}I_{q} \end{array}$$
[1']

In this case, the basic reproduction number would be given by

$$\begin{array}{rl} R_{0} & =\zeta \beta (1-q)N\{(1-p_{A})(1-x-y)+(1-\upsilon -p_{B})x+(I+\omega -p_{C})y\}\\ & \;\times \left\{\frac{1}{\delta +\gamma_{1}}+\frac{\theta }{\delta +\gamma_{3}}+\frac{\delta \pi }{\gamma_{2}(\delta +\gamma_{1})}\right\} \end{array}$$
[2']

This would allow to increase the realism for simulation.
